# Hydrogen peroxide priming modulates abiotic oxidative stress tolerance: insights from ROS detoxification and scavenging

**DOI:** 10.3389/fpls.2015.00420

**Published:** 2015-06-16

**Authors:** Mohammad A. Hossain, Soumen Bhattacharjee, Saed-Moucheshi Armin, Pingping Qian, Wang Xin, Hong-Yu Li, David J. Burritt, Masayuki Fujita, Lam-Son P. Tran

**Affiliations:** ^1^Department of Genetics and Plant Breeding, Bangladesh Agricultural UniversityMymensingh, Bangladesh; ^2^Department of Botany, University of BurdwanBardhaman, India; ^3^Department of Crop Production and Plant Breeding, College of Agriculture, Shiraz UniversityShiraz, Iran; ^4^Department of Biological Science, Graduate School of Science, Osaka UniversityToyonaka, Japan; ^5^School of Pharmacy, Lanzhou UniversityLanzhou, China; ^6^Gansu Key Laboratory of Biomonitoring and Bioremediation for Environmental Pollution, School of Life Sciences, Lanzhou UniversityLanzhou, China; ^7^Department of Botany, University of OtagoDunedin, New Zealand; ^8^Laboratory of Plant Stress Responses, Faculty of Agriculture, Kagawa UniversityTakamatsu, Japan; ^9^Signaling Pathway Research Unit, RIKEN Center for Sustainable Resource ScienceYokohama, Japan

**Keywords:** hydrogen peroxide, abiotic stress, oxidative stress, priming, stress tolerance

## Abstract

Plants are constantly challenged by various abiotic stresses that negatively affect growth and productivity worldwide. During the course of their evolution, plants have developed sophisticated mechanisms to recognize external signals allowing them to respond appropriately to environmental conditions, although the degree of adjustability or tolerance to specific stresses differs from species to species. Overproduction of reactive oxygen species (ROS; hydrogen peroxide, H_2_O_2_; superoxide, $O_{2}^{\cdot -}$; hydroxyl radical, OH^⋅^ and singlet oxygen, ^1^O_2_) is enhanced under abiotic and/or biotic stresses, which can cause oxidative damage to plant macromolecules and cell structures, leading to inhibition of plant growth and development, or to death. Among the various ROS, freely diffusible and relatively long-lived H_2_O_2_ acts as a central player in stress signal transduction pathways. These pathways can then activate multiple acclamatory responses that reinforce resistance to various abiotic and biotic stressors. To utilize H_2_O_2_ as a signaling molecule, non-toxic levels must be maintained in a delicate balancing act between H_2_O_2_ production and scavenging. Several recent studies have demonstrated that the H_2_O_2_-priming can enhance abiotic stress tolerance by modulating ROS detoxification and by regulating multiple stress-responsive pathways and gene expression. Despite the importance of the H_2_O_2_-priming, little is known about how this process improves the tolerance of plants to stress. Understanding the mechanisms of H_2_O_2_-priming-induced abiotic stress tolerance will be valuable for identifying biotechnological strategies to improve abiotic stress tolerance in crop plants. This review is an overview of our current knowledge of the possible mechanisms associated with H_2_O_2_-induced abiotic oxidative stress tolerance in plants, with special reference to antioxidant metabolism.

## Introduction

In plants the production of reactive oxygen species (ROS) is a common outcome of various metabolic reactions that occur in multiple sites within a plant cell. ROS like hydrogen peroxide (H_2_O_2_), superoxide ($O_{2}^{\cdot -}$), the hydroxyl radical (OH^⋅^) and singlet oxygen (^1^$O_{2}^{\cdot }$) are also produced as one of the earliest responses of plant cells to environmental stresses, and these ROS molecules can cause damage to a variety of biological processes (Halliwell, 2006; Gill and Tuteja, 2010; Das and Roychoudhury, 2014). In plants subjected to various abiotic stresses, such as salt, drought, chilling, heat and metal or metalloid stresses, ROS levels can rise significantly, leading to redox imbalance and oxidative stress (Hossain et al., 2010; Hasanuzzaman et al., 2011a,b; Hossain and Fujita, 2013; Mostofa and Fujita, 2013; Das and Roychoudhury, 2014; Mostofa et al., 2014a,b,c; Nahar et al., 2014). High ROS levels can result in extensive damage to proteins, DNA, and lipids, thereby affecting normal cellular functions, which can lead to permanent metabolic dysfunction and plant death (Anjum et al., 2015). To combat oxidative stress, plants have developed an elaborate system to control cellular ROS titer (Mittler et al., 2011). Surprisingly, plants have also evolved a way to exploit lower titer of ROS as signaling component to regulate wide variety of plant processes, including cell elongation, differentiation, morphogenesis and responses to environmental stress (Dat et al., 2000; Foreman et al., 2003; Tsukagoshi et al., 2010; Bhattacharjee, 2012).

In the last decade, H_2_O_2_ received considerable interest among the ROS and other oxygen-derived free radicals. H_2_O_2_, the result of two electron reduction via $O_{2}^{\cdot -}$ (the first step one electron reduction component), possesses the highest half-life (1 ms) of the ROS. A comparatively long life span and the small size of H_2_O_2_ molecules permit them to traverse through cellular membranes to different cellular compartments, facilitating signaling functions, including retrograde signaling (Apel and Hirt, 2004; Bienert et al., 2006; Maruta et al., 2012; Noctor et al., 2014). The signaling role of H_2_O_2_ is well established, particularly with reference to plant processes like stress acclimation, antioxidative defense, cell wall cross-linking, stomatal behavior, phytoalexin production, regulation of the cell cycle, and photosynthesis. So, the toxicity or danger associated with H_2_O_2_ on one hand and signaling cascades on other make it a versatile molecule whose concentration needs to be tightly controlled within plant cells (Petrov and Van Breusegem, 2012).

**FIGURE 1:**
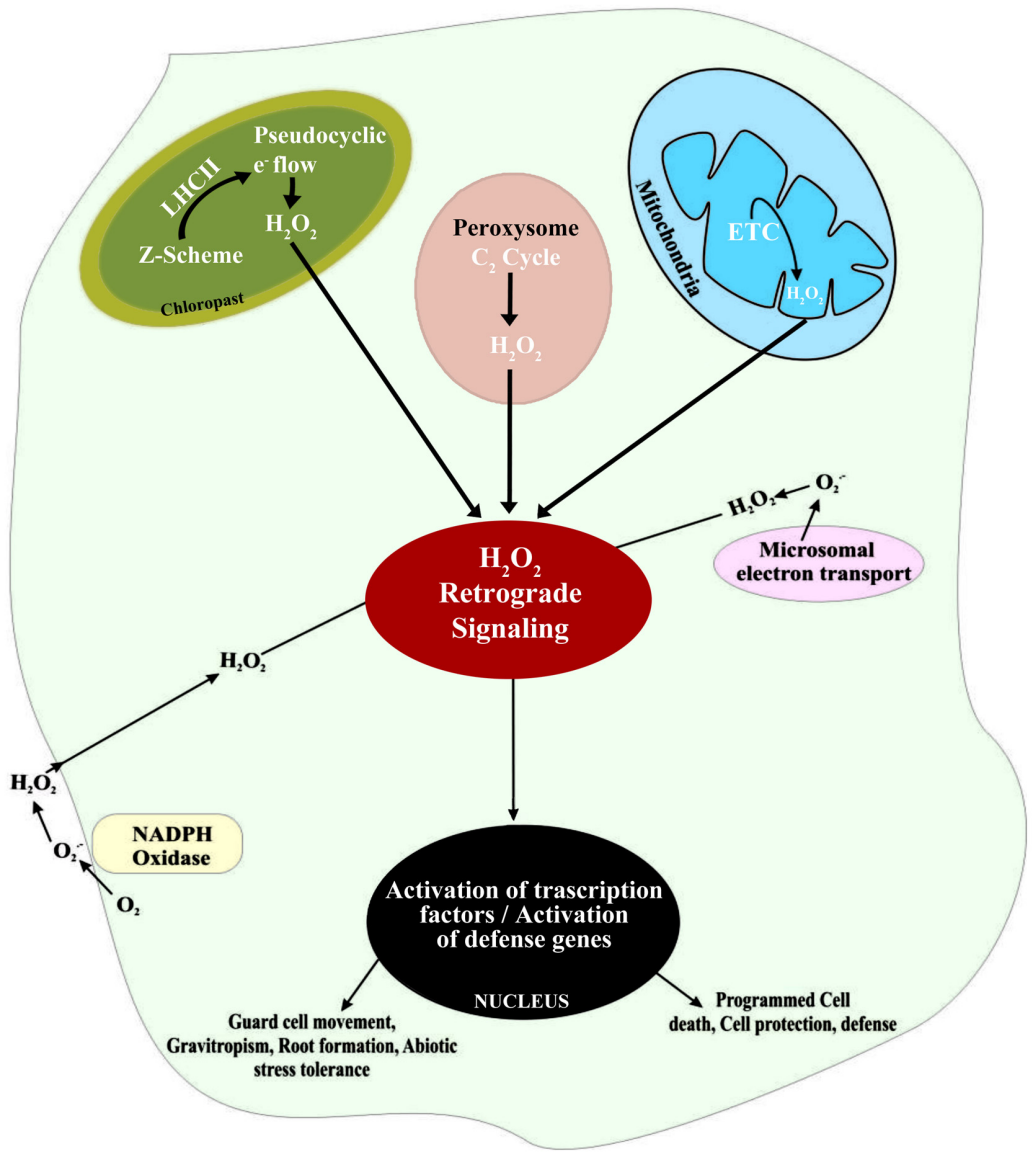
**Schematic representation of H_2_O_2_ generation in different intra- and extra-cellular sites and the subsequent signaling associated with the regulation of defense gene expression in plant cells**.

There are multiple sources of H_2_O_2_ in plant cells, including over-energization of electron transport chains (ETC) or redox reactions in chloroplasts or mitochondria, fatty acid oxidation, and photorespiration (**Figure 1**). Of these sources, the most significant is oxidation of glycolate in the peroxisome during the photosynthetic carbon oxidation cycle. In addition, the oxidative burst associated with part of hypersensitive response to pathogens also cause rapid increase in the concentration of H_2_O_2_ (Miller et al., 2010). One of the main sources of H_2_O_2_ is a class of cell membrane-bound NADPH-dependent oxidases that are similar to the respiratory burst oxidase homologs (RBOH). In plants, RBOH are in fact enzymes regulated by a class of Rho-like proteins called ‘ROP’s (Rho-related GTPases; Agrawal et al., 2003). Another class of enzymes, associated with the formation of H_2_O_2_, is the cell wall-associated peroxidases (Bolwell et al., 2002). Rates of H_2_O_2_ accumulation in peroxisomes and chloroplasts may be 30–100 times higher compared with H_2_O_2_ formation in mitochondria. Importantly, ROS formation in mitochondria does not vary significantly in presence or absence of light, since the total O_2_ consumption is less affected by light than TCA cycle activity. However, the formation of $O_{2}^{\cdot -}$ by electron transport systems can be influenced by light, if exposure to light affects alternative oxidase activity (Dutilleul et al., 2003). Alternative oxidases have been found to influence ROS generation and to be involved in determining cell survival under stressful conditions (Maxwell et al., 1999; Robson and Vanlerberghe, 2002).

The antioxidant systems that regulate H_2_O_2_ levels consist of both non-enzymatic and enzymatic H_2_O_2_ scavengers. Enzymes, such as catalase (CAT), ascorbate peroxidase (APX), glutathione peroxidase (GPX), glutathione *S*-transferases (GSTs), glutathione reductase (GR), and peroxyredoxin (Prx), and non-enzymatic compounds, like ascorbate (AsA), glutathione (GSH), α-tocopherol and flavonoids, are constantly involved in regulating the concentration of ROS, including H_2_O_2_ (Miller et al., 2010; Kapoor et al., 2015). In fact, both the production and scavenging of H_2_O_2_ in plant cells seem to be integrated in a network and are responsible for the ‘biological effect.’ The paradox of H_2_O_2_ physiology lies with its opposing activities; at higher concentrations it causes oxidative damage to important cellular metabolites, whereas at lower concentrations it initiates cell signaling (Gechev and Hille, 2005; Bhattacharjee, 2012). The redox imbalance associated with environmental stresses, such as salinity and extremes of temperature, increases the overall rate of metabolism and eventually up-regulates H_2_O_2_ production in plant cells (Bhattacharjee, 2012, 2013).

Priming (pre-treatment of seeds or plants by exposure to stressor or chemical compounds, making them more tolerant to later stress events) is potentially an important mechanism of induced resistance in plants against biotic stresses (Beckers and Conrath, 2007; Tanou et al., 2012; Borges et al., 2014). Recent studies have shown that priming can also modulate abiotic stress tolerance (Filippou et al., 2012; Hossain and Fujita, 2013; Mostofa and Fujita, 2013; Borges et al., 2014; Mostofa et al., 2014a,b,c; Nahar et al., 2014; Wang et al., 2014a). Despite the agronomic and ecological importance of priming, in-depth molecular mechanisms associated with priming in plants are still unknown (Conrath, 2011). Mounting evidence suggests that the initial exposure to chemical priming agents (such as H_2_O_2_, ABA, NO, SA etc.) renders plants more tolerant to abiotic stresses (Wang et al., 2010a; Hasanuzzaman et al., 2011a; Mostofa and Fujita, 2013; Mostofa et al., 2014a; Sathiyaraj et al., 2014; Teng et al., 2014). A number of studies on plants have demonstrated that the pre-treatment with an appropriate level of H_2_O_2_ can enhance abiotic stress tolerance through the modulation of multiple physiological processes, such as photosynthesis, and by modulating multiple stress-responsive pathways, such as the ROS and methylglyoxal (MG) detoxification pathways (Azevedo-Neto et al., 2005; Chao et al., 2009; Liu et al., 2010a; Xu et al., 2010; Wang et al., 2010a, 2014a; Ishibashi et al., 2011; Gondim et al., 2012, 2013; Hossain and Fujita, 2013). Although H_2_O_2_ is known to act as a signaling molecule, activating multiple defense responses that reinforce resistance to various environmental stresses in plants (Petrov and Van Breusegem, 2012), very little is known about the mechanisms by which plants perceive/sense H_2_O_2_ and how this sensing mechanism is coordinated within the developmental program of a plant. In this review, we summarize our current understanding of the possible mechanisms associated with H_2_O_2_-induced enhanced abiotic stress tolerance with special reference to ROS detoxifying/scavenging proteins and gene expression.

## Exogenous H_2_O_2_ and Abiotic Stress Tolerance

Many recent studies on plants have demonstrated that H_2_O_2_ is a key player in the signal transduction process associated with tolerance to abiotic and biotic stresses, and the induction of the stress cross-tolerance phenomena often observed in plants. A number of reports, discussed in more detail below, have shown that exogenous application of H_2_O_2_ can induce tolerance to salinity, drought, chilling, and high temperatures, and heavy metal stress, all of which cause elevated H_2_O_2_ production (Gong et al., 2001; Uchida et al., 2002; Azevedo-Neto et al., 2005; Chao et al., 2009; Liu et al., 2010a; Wang et al., 2010a, 2014a; Ishibashi et al., 2011; Gondim et al., 2012, 2013; Hossain and Fujita, 2013).

### Exogenous H_2_O_2_ and Salt Stress Tolerance

The salt stress-induced oxidative burst due to uncontrolled ROS accumulation has been well documented in plants. However, several recent studies on plants have demonstrated that pre-treatment with exogenous H_2_O_2_ can induce salt tolerance. Uchida et al. (2002) studied the effects of H_2_O_2_ and nitric oxide (NO) pre-treatments on oxidative stress in rice (*Oryza sativa*) plants under salt or heat stress. Their results showed that seedlings treated with low concentrations (<10 μM) of H_2_O_2_ or NO resulted in greener leaves and a higher photosynthetic activity than that of the control plants under conditions of salt or heat stress. It was also shown that pre-treatment induced increases in ROS scavenging enzyme activities and increased expression of genes encoding Δ′-pyrroline-5-carboxylate synthase, sucrose-phosphate synthase, and the small heat shock protein 26. Their findings indicate that NO and H_2_O_2_ act as signaling molecules that modulate heat and salt stress tolerance by regulating the expression of stress-related genes. In addition, Azevedo-Neto et al. (2005) found that supplementation of the nutrient solution with H_2_O_2_ induced salt tolerance in maize plants, by enhancing antioxidant metabolism and reducing lipid peroxidation in both leaves and roots. Wahid et al. (2007) reported that exogenous H_2_O_2_ improved salinity tolerance in *Triticum aestivum* when seeds were soaked in H_2_O_2_ (1–120 μM, 8 h) and subsequently grown in saline conditions (150 mM NaCl). H_2_O_2_ levels in the seedlings, arising from H_2_O_2_-treated seeds, were markedly lower when grown under saline conditions than control seedlings from seeds not treated with H_2_O_2_, and also exhibited better photosynthetic capacity. These results suggest that seedlings from H_2_O_2_-treated seeds had more effective antioxidant systems than found in untreated controls. Moreover, the H_2_O_2_ treatment appeared to improve leaf water relations, helped to maintain turgor, and improved the K^+^:Na^+^ ratio of salt stressed seedlings. H_2_O_2_ treatment also enhanced membrane properties, with greatly reduced relative membrane permeability (RMP) and lower ion leakage. Surprisingly, the expression of two heat-stable proteins (32 and 52 kDa) was also observed in H_2_O_2_ pre-treated seedlings. Fedina et al. (2009) reported that *Hordeum vulgare* seedlings pre-treated with H_2_O_2_ (1 and 5 mM) had higher rates of CO_2_ fixation and lower malondialdehyde (MDA) and H_2_O_2_ contents, following exposure to 150 mM NaCl for 4 and 7 days, when compared with seedlings subjected to NaCl stress only. In addition, the leaf Cl^-^ content of NaCl treated plants was considerably less in H_2_O_2_ pre-treated plants. The above findings indicate that H_2_O_2_ metabolism might be important for the induction of salt tolerance.

Gondim et al. (2010) evaluated the roles of H_2_O_2_ on the growth and acclimation of maize (*Zea mays*) triple hybrid (BRS3003) seedlings exposed to salinity stress, with three consecutive studies. In the first studies, H_2_O_2_ accelerated the percentage germination of seeds at 100 mM, but not at 500 mM H_2_O_2_. In second study, pre-treatment of seeds with H_2_O_2_ caused an up-regulation of APX and CAT activities after 30 h. In contrast, GPX activity was lower in seeds primed with H_2_O_2_ for 12, 24, 30, 36, and 42 h as compared with the seeds primed with water only. The activity of SOD was not affected by pre-treatment of seeds with H_2_O_2_, except for the 24 h pre-treatment. In the third experiment, seeds were pre-treated by soaking in 100 mM H_2_O_2_ for 36 h, or in distilled water (DW), and then grown in a culture solution with or without salt stress (80 mM NaCl). Their findings showed that priming of seeds with H_2_O_2_ increased seedling tolerance to salinity, with seedlings demonstrating improved growth rates. The differences in the levels of antioxidant enzyme activities detailed above may explain the higher salinity tolerance of seedlings from seeds pre-treated with H_2_O_2_. In addition, Li et al. (2011) reported that exogenously applied H_2_O_2_ (0.05 μM) reduced the MDA content, enhanced the GSH content and increased the activities of APX, CAT, SOD, and POD in wheat seedlings under salt stress. A similar response in *Suaeda fruticosa* (a halophyte) was also found, indicating that cellular defense antioxidant mechanisms are enhanced by the exogenous application of H_2_O_2_ (Hameed et al., 2012). Up-regulation of the activities of CAT and SOD following the exogenous application of H_2_O_2_ (0.5 mM) was also observed in oat (*Avena sativa*) plants under salt stress (Xu et al., 2008). Similarly, Gondim et al. (2012) found that foliar H_2_O_2_ priming was effective in minimizing salt stress in maize and analysis of the antioxidant enzymes CAT, GPOX, APX, and SOD revealed that the H_2_O_2_ foliar spray increased the activities of all of these enzymes. CAT was found to be the most highly responsive of the above enzymes to H_2_O_2_, with high activities observed (48 h) after treatment, while GPX and APX responded much later (240 h after treatment). Lower MDA levels were also detected in maize plants with higher CAT activities, which may have resulted from the H_2_O_2_ detoxifying function of this enzyme. In addition, Gondim et al. (2013) studied the influence of exogenous H_2_O_2_ application on AsA and GSH metabolism, relative chlorophyll content, relative water content (RWC), and gas exchange, in *Zea mays* grown under salinity. Photosynthesis and transpiration, stomatal conductance, and intercellular CO_2_ concentrations all declined in plants under salt stress; however, the negative impact of salt stress was not as great in plants sprayed with H_2_O_2_. In addition, H_2_O_2_-sprayed plants had higher RWCs, relative chlorophyll contents and lower leaf H_2_O_2_ accumulation, which correlated positively with improved gas exchange, compared with control plants under conditions of NaCl stress. The non-enzymatic antioxidants AsA and GSH did not appear to play any obvious roles as ROS scavengers in this study. The authors of the above study concluded that salt tolerance of maize plants, brought by pretreatment of leaves with H_2_O_2_, was due to less H_2_O_2_ accumulation and to maintenance of the leaf RWC and chlorophyll contents. These characteristics allowed higher photosynthesis and improved growth of maize plants under salt stress. In addition to these findings, Ashfaque et al. (2014) conducted an experiment to study the role H_2_O_2_ played in mitigating salt stress in wheat (*Triticum aestivum* L.) plants. Treatment of plants with H_2_O_2_ positively influenced plant growth under saline and non-saline conditions. The application of 50 or 100 μM H_2_O_2_ reduced the severity of salt stress, with reductions in both Na^+^ and Cl^-^ ion levels and an increase in proline content and in N assimilation. Improved water relations, increased levels of photosynthetic pigments and greater growth rates were also observed in H_2_O_2_ under salt stress when compared with untreated plants. Under non-saline conditions application of H_2_O_2_ also improved all the parameters detailed above. Treatment with 100 μM H_2_O_2_ provided maximal protection for wheat plants grown under non-saline conditions and also alleviated the effects of salt stress in plants grown under saline conditions. Recently, Sathiyaraj et al. (2014) found that *Panax ginseng* seedlings treated with 100 μM H_2_O_2_ for 2 days showed enhanced salinity tolerance and increased activities of APX, CAT, and guaiacol peroxidase. Other oxidative parameters such as MDA levels and endogenous H_2_O_2_ and $O_{2}^{\cdot -}$ levels were lower in H_2_O_2_ treated salt-stressed seedlings. Seedling dry weight, and chlorophyll and carotenoid contents were also greater in H_2_O_2_ treated seedlings than in untreated controls, when seedlings were subjected to salt stress. The above findings demonstrate that H_2_O_2_ priming can induce tolerance to salinity in plants by modulating physiological and metabolic processes such as photosynthesis, proline accumulation and ROS detoxification, and that this ultimately leads to better growth and development. Importantly, ROS metabolism also plays a pivotal role in the development of stress and cross stress tolerance.

### Exogenous H_2_O_2_ and Drought Stress Tolerance

Drought stress is widely thought to induce oxidative stress by increasing the levels of H_2_O_2_ and singlet oxygen (de Carvalho, 2013). However, Jing et al. (2009) investigated the capacity of H_2_O_2_ priming to promote drought tolerance in Cucumber plants. Drought stress resulted in cucumber plants with round chloroplasts, and indistinct chloroplast membranes and thylakoids. While H_2_O_2_ priming did not change chloroplast ultrastructure, priming did increase the activities of the antioxidant enzymes SOD, CAT, GPOX, APX, DHAR, DHAR, GR, and the levels of AsA and GSH, resulting in lower levels of MDA, H_2_O_2_ and $O_{2}^{\cdot -}$. The authors of this study concluded that by increasing antioxidant capacity H_2_O_2_ priming reduced the accumulation of ROS in treated plants, and alleviated some of the membrane damage found in the chloroplasts of plants under drought stress. In a similar study Ishibashi et al. (2011) showed that spraying plants with H_2_O_2_ could alleviate the symptoms of drought stress in soybean. The RWC content, photosynthetic rate and stomatal conductance of drought-stressed leaves in plants sprayed with H_2_O_2_ were all higher than in leaves sprayed with DW. In contrast to spraying with DW, spraying with H_2_O_2_ caused an increase in the expression of *galactinol synthase* (*GolS*) and *d-myo-inositol 3-phosphate synthase 2* (*GmMIPS2*) genes, which are responsible for the synthesis of oligosaccharides. These findings indicated that H_2_O_2_ spraying enabled soybean plants to avoid drought stress by helping to maintain leaf water levels, and that leaf water retention was probably due to increased oligosaccharide biosynthesis rather than rapid stomatal closure. Abass and Mohamed (2011) also studied the effects of priming seeds with H_2_O_2_ on the drought tolerance of common bean seedlings (*Phaseolus vulgaris* L.). A significant decrease in plant growth parameters, photosynthetic pigments, and the total carbohydrate content was observed in response to drought stress. In contrast, a significant increase in compatible solutes, polyamine and antioxidant levels, and abscisic acid (ABA) contents were observed in plants in response to drought stress. H_2_O_2_-priming of seeds enhanced all of the above parameters in seedlings grown under drought conditions when compared with the seedlings of water-treated seeds. The above findings suggest that H_2_O_2_ could trigger the activation of defense mechanisms, including increased levels of antioxidants, which then persist in developing seedlings and help to alleviate damage and improve plant growth and performance under drought. Liao et al. (2012) studied the beneficial roles of exogenous NO and H_2_O_2_ in marigold (*Tagetes erecta* L.) adventitious root formation in response to drought. NO or H_2_O_2_ treatment reduced the damage to mesophyll cell ultrastructure caused by drought stress. NO or H_2_O_2_ treatment also increased leaf chlorophyll contents, chlorophyll fluorescence parameters (Fv/Fm, ΦPS II, and qP), and hypocotyl soluble carbohydrate and protein contents, while reducing starch contents. These findings demonstrate that NO or H_2_O_2_ can protect mesophyll cell ultrastructure from damage, improve the photosynthetic performance of leaves and mitigate the negative effects of drought stress, by enhancing nitrogen and carbohydrate accumulation. Recently, Hossain and Fujita (2013) examined the potential biochemical mechanisms of H_2_O_2_ priming-induced drought tolerance in mustard (*Brassica juncea* L.) seedlings by investigating ROS scavenging and MG metabolism. Eight-day-old seedlings were pre-treated with a low concentration (50 μM) of H_2_O_2_ for 24 h prior to the imposition of drought stress for 48 h. H_2_O_2_ priming enhanced cell membrane stability in leaf tissues under drought stress, by reducing tissue MDA contents. The levels of endogenous H_2_O_2_, in H_2_O_2_ pre-treated, drought stressed-seedlings were markedly lower than that of seedlings subjected to drought stress without H_2_O_2_ pre-treatment. Lower activities of APX, CAT, and Gly II were observed in response to drought stress, whereas DHAR, GPX, and Gly I activities significantly increased. AsA, GSH, and GSSG levels increased significantly, whereas the GSH/GSSG ratio decreased in drought-stressed seedlings. Surprisingly, H_2_O_2_ pre-treated drought-stressed seedlings maintained significantly higher APX, GR, CAT, GST, and Gly II activities, as well as a higher GSH/GSSG ratio compared with seedlings under drought only. These results show that H_2_O_2_ priming can activate both ROS and MG detoxification pathways and modulate the tolerance of seedlings to water deficit (Hossain and Fujita, 2013). Ashraf et al. (2014) investigated the beneficial roles of exogenous H_2_O_2_ on drought stress tolerance in maize. Maize seedlings were pre-treated with different concentrations of H_2_O_2_ and grown under conditions of water stress. Higher germination percentages were found in seeds soaked in 140 mM H_2_O_2_. Drought led to a sharp decrease in photosynthetic pigments, whereas the levels of H_2_O_2_, lipid peroxidation and AsA increased. The activities of CAT, SOD, and POX rapidly increased. Importantly, the 140 mM H_2_O_2_ treatment reduced photosynthetic pigment degradation and lipid peroxidation and increased the activities of antioxidant enzymes and AsA levels. The beneficial influence of exogenous H_2_O_2_ treatments have also been observed in plants under osmotic stress. Liu et al. (2010a) studied the effects of exogenous H_2_O_2_ on osmotic stress-induced alterations in the ultra-structures of chloroplasts and mitochondria in two cucumber (*Cucumis sativus* L.) varieties. Osmotic stress caused the degradation of chloroplast and mitochondrial membranes in both cucumber genotypes and increased MDA levels. Osmotic stress and exogenous H_2_O_2_ both increased MnSOD, GPX, CAT, GPOX, APX, GR, MDHAR, DAHR activities and levels of the antioxidants AsA and GSH. The combined effects of osmotic stress and exogenous H_2_O_2_ resulted in the highest antioxidant levels in both cucumber ecotypes. Liu et al. (2010a) proposed that pre-treatment with H_2_O_2_ increased antioxidant levels in the leaves of cucumbers, thereby decreasing MDA levels, and protecting the ultrastructure of most chloroplasts and mitochondria in plants under osmotic stress. Terzi et al. (2014) also found that exogenous H_2_O_2_ (10 mM) pre-treatment induced osmotic stress tolerance in maize (*Zea mays* L.) seedlings. H_2_O_2_ treatment caused a decrease in MDA levels and stomatal conductance, whereas an increase in endogenous H_2_O_2_, leaf water potential, ABA concentration, and metabolite levels, including soluble sugars, proline, and polyamines, were observed. Osmotic stress caused a decline in leaf water potential and stomatal conductance, but the levels of MDA, H_2_O_2_, metabolite levels and the ABA content increased. Importantly, H_2_O_2_ pretreated osmotical stressed seedlings showed improved water status and stomatal conductance, as well as accumulation of MDA, H_2_O_2_, ABA, and metabolites. These results demonstrate that H_2_O_2_ pre-treatment induces osmotic stress tolerance by increasing soluble sugar, proline, and polyamine levels.

### Exogenous H_2_O_2_ and Chilling Stress Tolerance

The positive role of exogenous H_2_O_2_ in modulating low temperature stress tolerance has been well documented. Prasad et al. (1994a,b) reported that addition of H_2_O_2_ modulated chilling tolerance, due to a transient increase in H_2_O_2_-activated acclimation mechanisms. The authors suggested that H_2_O_2_ has dual effects on maize plants during acclimation to chilling; it serves as a signal to induce the synthesis of ROS-scavenging enzymes, and in non-acclimated seedlings it accumulates to higher levels and acts as a destructive agent. Additionally, it was reported that both H_2_O_2_ and SA could mediate the induction of protective mechanisms against abiotic stresses. SA pre-treatment induced an increase in H_2_O_2_ concentrations that in turn triggers an increase in antioxidant enzyme activities and eventually leads to higher tolerance to chilling stress in maize seedlings (Janda et al., 1999). Likewise, H_2_O_2_ and SA were involved in the signal transduction pathway leading to acclimation during heat stress in mustard (Dat et al., 1998). Yu et al. (2003) showed that a transient oxidative shock, induced by exogenous H_2_O_2,_ effectively increased chilling tolerance in mung bean (*Vigna radiata* L. cv. V3327) seedlings. Seedlings pre-treated with 200 mM H_2_O_2_ had increased survival rates (from 30 to 70%) and lowered EL (86 to 21%). Importantly, the endogenous level of H_2_O_2_ was not affected by exogenous application of H_2_O_2_. Surprisingly, exogenous H_2_O_2_ repressed the stimulation of ROS detoxifying enzymes APX and CAT; however, GSH levels increased significantly under both chilling and control conditions. Pre-treatment of mung bean plants with both ABA and H_2_O_2_ showed no synergistic effect on GSH content. The authors concluded that H_2_O_2_-mediated chilling tolerance in mung bean plants might be mediated by an increase in GSH content that is independent of ABA. Supporting this finding, Hung et al. (2007) showed that H_2_O_2_ pre-treatment induced chilling tolerance in chilling sensitive mung bean seedlings (*V. radiata* L. Cv Tainan Number 5). Seedlings pre-treated with 200 mM H_2_O_2_ or cold-acclimated (10°C for 48 h in the light) showed lower electrolyte leakage (EL) compared to seedlings subjected to chilling stress (4°C for 36 h) without H_2_O_2_ treatment or cold-acclimation. Chilling tolerance induced by H_2_O_2_ appeared to depend on the accumulation of GSH, as tolerance could be reversed by pretreatment with buthionine sulfoximine (BSO). In contrast, tolerance induced by cold-acclimation was neither accompanied by the accumulation of GSH nor reversed by BSO, suggesting that there are at least two independent mechanisms for developing chilling tolerance. Wang et al. (2010a) studied the effects of foliar pre-treatment with H_2_O_2_ in modulating chilling stress tolerance of mascarene grass (*Zoysia tenuifolia*) and manilagrass (*Zoysia matrella*). Pre-treatment with H_2_O_2_ (10 mM) was found to modulate chilling (7°C/2°C, day/night) stress tolerance as indicated by lower MDA and EL levels and higher protein contents. Pre-treatment significantly increased the activities of APX, GPX, and CAT in *Zoysia matrella* and APX, GR, and POD activities in *Zoysia tenuifolia*, indicating that H_2_O_2_ acts as a signaling molecule and modulates the metabolic responses associated with ROS-induced damage caused by chilling. Importantly, optimal pre-treatments reduced any increases in H_2_O_2_ levels, improved chilling tolerance, and increased CAT, POD, APX, GR, and GPX activities. Therefore, antioxidative enzymes are likely to be important factors for the acquisition of chilling tolerance in both *Zoysia* cultivars. Moussa and Mohamed (2011) showed that priming of pea seeds with H_2_O_2_ or NO significantly enhanced drought induced oxidative stress tolerance. Seeds were pre-treated with 70 mM H_2_O_2_ or 10 μM sodium nitroprusside (a NO donor). Seeds pre-treated pea seedlings have less ROS-induced damage, accelerated proline synthesis and enhanced total chlorophyll and carotenoid contents, increased photosynthetic activity, and increased growth when subjected to osmotic stress. Drought stress reduced the activities of APX, GPX, and CAT, and caused an overproduction of $O_{2}^{\cdot -}$ in the leaves of pea plants, which in turn increased MDA levels and reduced photosynthetic performance. Pre-treatment with SNP or H_2_O_2_ modulated the activities of antioxidant enzymes, limited $O_{2}^{\cdot -}$ production, and inhibited membrane peroxidation under drought stress, which indicated an enhanced operation of antioxidant systems. Moreover, after H_2_O_2_ or SNP pre-treatment seedlings had enhanced membrane stability as revealed by a lower MDA contents. The increased production of antioxidants in seedlings from seeds pre-treated with H_2_O_2_ or SNP persisted for some time, alleviating ROS-induced impairment and modulating the physiological characters associated with drought tolerance of seedlings. Recently, İşeri et al. (2013) investigated whether exogenous H_2_O_2_ application could influence the short-term cold responses of tomato plants and induce acclimation. Pre-treatments were performed by immersing roots into 1 mM H_2_O_2_ solution for 1 h and then transferring the seedlings to the soil (acclimated group). Cold stress (3°C for 16 h) caused a significant reduction in the RWC of control and non-acclimated groups when compared with unstressed plants. H_2_O_2_ promoted the maintenance of a higher RWC under stress. Anthocyanin levels in the leaves of acclimated plants under cold stress were significantly higher than those of unstressed control and non-acclimated plants. High MDA levels demonstrated low temperature induced oxidative damage in control and non-acclimated plants. MDA levels in acclimated plants remained similar to those of unstressed plants, which demonstrated that the H_2_O_2_ acclimation process protected the cells against cold induced lipid peroxidation. In addition, H_2_O_2_ acclimation caused proline accumulation in roots under cold stress and APX activity in the roots of cold-stressed and -unstressed H_2_O_2_-acclimated plants increased when compared with control and non-acclimated plants, with the highest increase in the roots of acclimated plants under cold stress. CAT levels in the roots of acclimated plants also increased, whereas levels remained unchanged in unstressed plants. Endogenous H_2_O_2_ levels increased significantly in the roots of control and non-acclimated plants under cold stress. In contrast, the H_2_O_2_ content of the roots of acclimated plants was significantly lower than that of control and non-acclimated plants under cold stress. These results demonstrate that H_2_O_2_ significantly enhances oxidative stress responses by elevating the antioxidant status of tomato plants.

### Exogenous H_2_O_2_ and Heat Tolerance

Like other abiotic stresses endogenous levels of H_2_O_2_ increase in heat stressed plants (Hossain et al., 2013a,b; Mostofa et al., 2014b) and exogenous pre-treatments haves been found to increase the heat tolerance of plants. Kang et al. (2009) reported that H_2_O_2_ pre-treatments increased the activities of APX and glucose-6-phosphated dehydrogenase (G6PDH) in cucumber and tomato seedlings, and induced tolerance to heat stress. Bhattacharjee (2012) reported that H_2_O_2_ enhanced tolerance of two rice cultivars differing in salt tolerance (SR 26B, salt-tolerant; Ratna, salt-sensitive cultivar) to heat- or chilling-induced oxidative stress. Salt or drought stress results in significant increases in lipid peroxidation and protein oxidation, along with concomitant increases in the accumulation of ROS ($O_{2}^{\cdot -}$and H_2_O_2_) and a reduction in antioxidant defenses (assessed in terms of total thiol content and the activities of SOD, CAT, APX, and GR) in both the seedlings of salt-sensitive Ratna and salt-tolerant SR 26B cultivars. Imbibitional treatment with low concentrations of H_2_O_2_ reduced oxidative damage to newly assembled membrane systems caused by heat and chilling stress in the seedlings of both cultivars of rice (Ratna and SR 26B). Imbibitional H_2_O_2_ treatment also caused an increase in antioxidant defenses (activities of SOD, CAT, APX, GR, and total thiol content) in the heat and chilling stressed seedlings and caused a significant improvement in the early growth performances of both cultivars. Better responses to H_2_O_2_-mediated acclamatory performances and restoration of redox-homeostasis under extremes of temperature were noted for the salt-sensitive rice cultivar Ratna compared with the salt-tolerant SR 26B. In general, these results suggest a significant role for an ‘inductive pulse’ of H_2_O_2_ in acclimatizing plants to adverse temperatures, by helping to maintain redox-homeostasis and mitigating oxidative membrane, protein and lipid damage during the recovery phase of the post-germination event. Wang et al. (2014a) studied the beneficial roles of exogenous H_2_O_2_ in modulating heat stress tolerance in turfgrass species. Ryegrass (*Lolium perenne* cv. Accent) and tall fescue (*Festuca arundinacea* cv. Barlexas) were sprayed with 10 mM H_2_O_2_ before they were exposed to heat stress (38/30°C, day/night) and compared with plants maintained at control temperatures (26/15°C, day/night). Before being subjected to heat stress seedlings treated with H_2_O_2_ were found to have increased activities of POD, CAT, APX, GR, and GPX, as well as larger AsA and GSH pools. Importantly the ratio of GSH/GSSG was also lower. Under heat stress H_2_O_2_ pre-treated seedlings showed lower oxidative damage and H_2_O_2_ levels, and increased activities of APX, GR, GST, and GPX. These results indicated that H_2_O_2_ could up-regulate the antioxidant defense systems that ultimately lead to improved thermotolerance in turfgrass species.

### Exogenous H_2_O_2_ and Heavy Metal Stress

Excessive production of ROS, especially H_2_O_2_, in response to heavy metal exposure has been widely observed in plants (Hossain et al., 2010; Mostofa and Fujita, 2013; Mostofa et al., 2014a). H_2_O_2_ priming has also been found to increase tolerance of plants to heavy metals. Hu et al. (2009) showed H_2_O_2_ pre-treatment induced Cd tolerance in rice (*O. sativa*). Cd stress led to a significant decrease in both the length and biomass of roots and shoots. However, pre-treatment with 100 μM H_2_O_2_ for 1 day mitigated Cd stress and increased the levels of the antioxidant enzymes (SOD, CAT, GPX, APX, and GST), as well as elevated the levels of GSH and AsA. Consequently, the levels of MDA and H_2_O_2_ declined and the growth rates of the seedlings improved. H_2_O_2_ pre-treatment also decreased the Cd concentration found in the shoots, and lowered the shoot:root Cd ratio, indicating that H_2_O_2_ may affect Cd distribution in rice seedlings. Improved Cd tolerance was thought to be partly due to enhanced antioxidant metabolism that effectively prevented the increase in ROS levels under Cd stress. Higher Cd sequestration in root tissue may also contribute to the decline in Cd translocation. Chao et al. (2009) investigated the role of GSH in modulating heavy metal (Cd) stress tolerance of rice seedlings. Seedlings treated with either a heat shock or H_2_O_2_ showed a significant increase in leaf GSH levels. Treatment with exogenous GSH under non-heat stress conditions, which also resulted in an increase in GSH levels in leaves, also enhanced the Cd tolerance of rice seedlings. Pre-treatment of seedlings with an inhibitor of GSH synthesis inhibited the increase in GSH levels caused by heat shock or H_2_O_2_ treatment and caused a reduction Cd tolerance. Importantly, the negative effects of BSO could be reversed by the addition of GSH. A time-course analyses of heat stress in rice seedlings demonstrated that the accumulation of H_2_O_2_ preceded the increase in GSH. This finding suggests that early augmentation of H_2_O_2_ levels during heat shock treatments acts as a signal to modulate GSH biosynthesis and to protect rice plants from Cd-induced damage. Xu et al. (2010) reported the H_2_O_2_-induced up-regulation of AsA and GSH metabolism-induced Al tolerance in wheat seedlings. Al stress resulted in higher $O_{2}^{\cdot -}$ and H_2_O_2_ contents, greater MDA levels, programmed cell death, and inhibited root growth in both rice genotypes. The activities of CAT, POD, SOD, GPX, GR, MDHAR, and DHAR and the levels of GSH and AsA increased in response to Al stress. However, H_2_O_2_-primed Al-stressed seedlings showed higher activities of GPX, CAT, POD, MDHAR, DHAR, and GR, and higher AsA and GSH contents as well as a more favorable redox state than seedlings subjected to Al-stress only. Notably, a large increase in ROS detoxifying enzyme activities was observed in the Al-susceptible genotype as compared with the resistant genotype. H_2_O_2_ pre-treatment increased the tolerance of plants to Al-induced oxidative stress by increasing level of GSH and AsA and the activities of enzymes involved in their metabolism. Bai et al. (2011) studied the effects of H_2_O_2_ pretreatment on Cd tolerance and translocation by utilizing two rice genotypes (N07-6 and N07-63) with contrasting Cd tolerance. Cd stress (50 μmol/L) led a sharp decline in seedling growth and increased production of MDA, GSH, NPT, and phytochelatins (PCs), as well as GST activity. H_2_O_2_ pre-treatment further improved Cd stress tolerance by increasing the levels of NPT, GSH, and PCs, and the activity of GST in roots. The increase was greater in N07-63 as compared with the N07-6.

Chou et al. (2012) investigated the involvement of H_2_O_2_ in heat-shock induced Cd tolerance, in relation to the activities of ROS detoxifying enzymes (APX and GR) in rice plants. Heat-shock treatments increased the content of H_2_O_2_ before increases in the activities of APX and GR were observed in rice leaves. Importantly, heat-shock induced *OsAPX2* gene expression was associated with heat shock induced increases in APX activity. Upon imposition of Cd stress the H_2_O_2_ content and the activities of APX and GR increased, but the increase was less than that observed in seedlings subjected to Cd-stress without heat pre-treatment. The authors concluded that H_2_O_2_ is involved in the regulation of heat shock and Cd-induced increases in the activities of GR and APX in rice leaves, and thus cross-tolerance in rice plants. Yildiz et al. (2013) studied the ameliorating effects of H_2_O_2_ on chromium (Cr) toxicity in canola (*B. napus* L.) plants in relation to thiol content, lipid peroxidation, antioxidant enzyme activities and the growth and chlorophyll content, as well as the levels of a metallothionein protein (BnMP1). Cr stress (50 μM) significantly reduced plant growth, which was accompanied by increased lipid peroxidation and a decrease in the chlorophyll content of the leaves. H_2_O_2_ pre-treatment, enhanced plant growth reduced the MDA levels and promoted higher levels of pigments. Additionally, the accumulation of Cr was higher in the aerial parts of the H_2_O_2_-pretreated seedlings. Increased thiol levels were observed under Cr stress and were further enhanced by H_2_O_2_ pre-treatment. POD and SOD activities increased in response to Cr stress, whereas the activities of CAT and APX decreased. H_2_O_2_ pre-treatment caused an increase in the activities of APX and POD in response to Cr stress, but CAT and SOD activities remained unchanged. *BnMP1* expression analysis showed enhanced expression after 1 day of treatment, and then a decrease after 7 days exposure to Cr. In contrast, after 7 days exposure to Cr, H_2_O_2_-pre-treated seedlings showed a less decrease in *BnMP1* expression as compared with the seedlings subjected to Cr stress only. These findings indicate that H_2_O_2_ may act as a signal that triggers defense mechanisms that in turn protect plants from ROS-induced damage caused by exposure to Cr. In addition, the roles of exogenous H_2_O_2_ on plant growth, water status, mineral ion content, proline content, and total sugar and soluble protein contents were evaluated in maize leaves exposed to copper (Cu) stress (0.5 mM Cu) by Guzel and Terzi (2013). Cu stress resulted in a decrease in leaf water potential, ionic concentration (Na^+^, K^+^, Ca^+^, Mg^2+^) and protein levels, but increases in the content of proline content and total soluble sugars, when compared with controls. Importantly H_2_O_2_-pre-treated seedlings showed an increase in growth, water content, mineral concentration, proline content, total soluble sugar and soluble protein contents when compared with control plants. A greater increase was also observed in the proline and total sugar contents of the Cu+H_2_O_2_ group of plants compared with the Cu stress alone group. These findings suggest that exogenous H_2_O_2_ can increase dry matter production and mineral ion distribution in maize seedlings. Additionally, osmotic regulation might be involved in the alleviation of Cu toxicity of maize leaves caused by pre-treatment of H_2_O_2_.

### Exogenous H_2_O_2_ and Multiple Stress Tolerance

The possible involvement of H_2_O_2_ in heat-induced cross-adaptation to salinity, drought, chilling and heat stress was studied by Gong et al. (2001) in two cultivars of maize differing in their stress tolerance. A heat-shock pre-treatment (42°C, 4 h) following 4-h recovery at a temperature of 28°C considerably enhanced the survival of seedlings, reduced the leakage of electrolyte from the roots and the loss of coleoptile vitality of seedlings after stress was imposed. Importantly, the heat-shock pre-treatment produced an H_2_O_2_ peak in the maize seedlings. The accumulation of H_2_O_2_ lead to the generation of cross-tolerance, indicating that an early short-lived increase in endogenous H_2_O_2_ is essential for the induction of cross-adaptation, by triggering increased expression of genes encoding ROS detoxification and therefore increasing the activities of antioxidant enzymes. Hence H_2_O_2_ could have a signaling role in inducing cross-tolerance in maize seedlings.

## H_2_O_2_-Induced Abiotic Stress Tolerance and a Possible Biochemical and Molecular Basis

**FIGURE 2:**
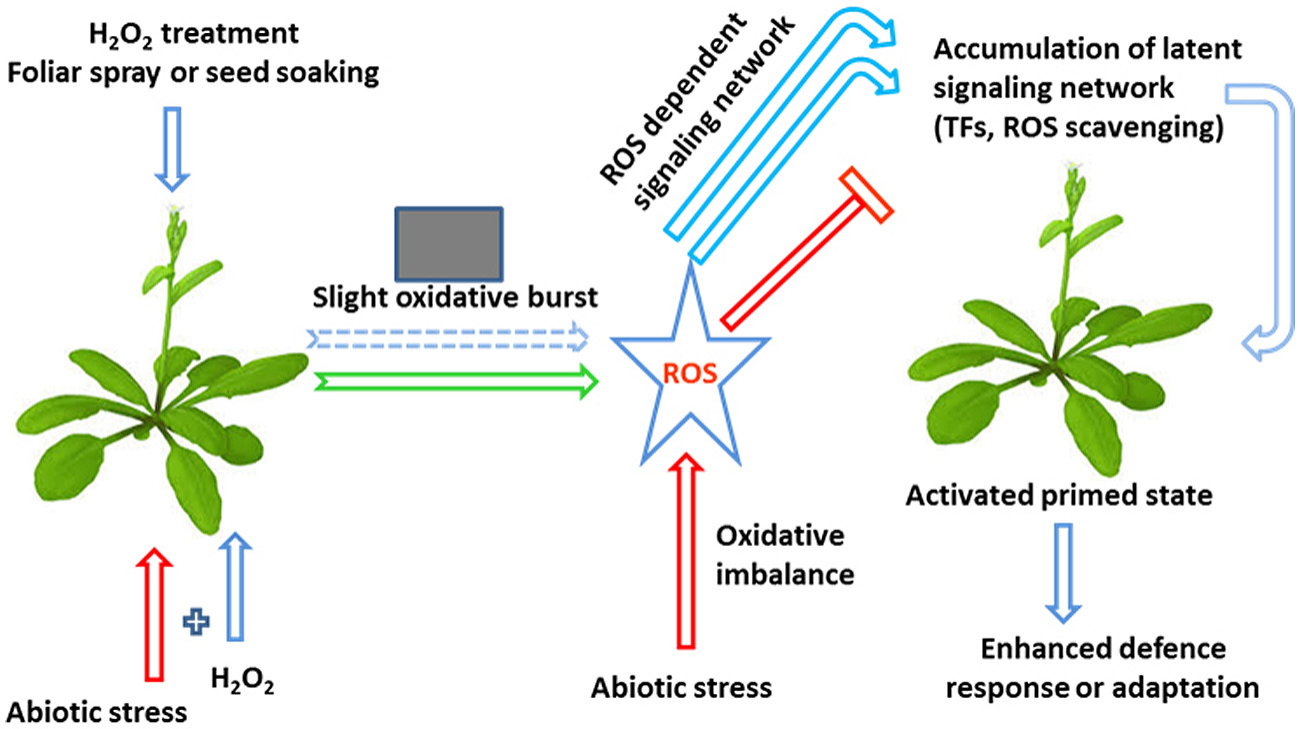
**A hypothetical model of the influence of H_2_O_2_ on plant defense mechanisms associated with abiotic stresses**. H_2_O_2_ treatment is capable of inducing abiotic stress tolerance through the development of a small oxidative burst. This burst subsequently activates a ROS-dependent signaling network, thereby enhancing the accumulation of latent defense proteins, such as ROS-scavenging enzymes and transcription factors (TFs), resulting in a primed state and an enhanced stress response (modified from Borges et al., 2014).

Recent studies have shown that H_2_O_2_ originating in the same cell can induce two dissimilar kinds of responses: one that depends on the subcellular sites of H_2_O_2_ production and the other integrating H_2_O_2_ signals independently of the subcellular site of production. Several studies have shown that H_2_O_2_ is a part of the retrogate signaling mechanism, from mitochondria or chloroplasts that activate the expression of nuclear-encoded stress-responsive genes. The aforementioned evidence clearly demonstrates that H_2_O_2_ priming modulates abiotic stress tolerance in plants by modulating ROS and MG detoxification and scavenging (Gong et al., 2001; Uchida et al., 2002; Yu et al., 2003; Xu et al., 2008, 2010; Hu et al., 2009; Jing et al., 2009; Gondim et al., 2010, 2012; Liu et al., 2010a; Wang et al., 2010a; Li et al., 2011; Bhattacharjee, 2012; Chou et al., 2012; Zhang et al., 2012; Hossain and Fujita, 2013; Ashraf et al., 2014; Sathiyaraj et al., 2014; Wang et al., 2014a), by enhancing the expression of heat shock proteins (Uchida et al., 2002; Wahid et al., 2007), by enhancing GSH and AsA biosynthesis (Yu et al., 2003; Hung et al., 2007; Chao et al., 2009; Xu et al., 2010; Bai et al., 2011; Wang et al., 2014a), by enhancing proline biosynthesis (Uchida et al., 2002; Guzel and Terzi, 2013; İşeri et al., 2013; Ashfaque et al., 2014; Sathiyaraj et al., 2014; Terzi et al., 2014), by enhancing photosynthesis (Wahid et al., 2007; Ishibashi et al., 2011; Moussa and Mohamed, 2011; Liao et al., 2012; Gondim et al., 2013), by enhancing ABA biosynthesis (Abass and Mohamed, 2011; Terzi et al., 2014) and by regulating multiple stress responsive pathways and genes. Based on the above findings we propose a hypothetical model that summarizes the possible mode of action of H_2_O_2_ in planta (**Figure 2**). It is speculated that H_2_O_2_ priming induces a mild oxidative stress by disruption of cellular ROS homeostasis, from which develops a ROS-dependent signaling network and induces the accumulation of latent defense proteins such as ROS-scavenging enzymes and transcription factors (TFs), among others, resulting in a primed state and enhanced stress responses. Another notable effect of H_2_O_2_ is its capacity to induce the expression of TFs and genes responsible for osmolyte synthesis, e.g., proline and betaine, and activate phosphorylation cascades using mitogen-activated protein kinases (MAPKs). In the following section we will critically discuss the perception of extracellular and intracellular H_2_O_2_ by plants and how this signal transduces and modulates the abiotic stress tolerance of plants.

### H_2_O_2_ as a Signaling Molecule Involved in Stress Tolerance

#### Sensing of H_2_O_2_

H_2_O_2_ can act as a signaling molecule via chemical reactions with targeted amino acids that can lead to peptide/protein modifications. It is now well established that GSH and protein cysteine (Cys) residues are particularly well suited for reactions with oxidants such as H_2_O_2_ (D’Autréaux and Toledano, 2007; Munné-Bosch et al., 2013). Cys residues in proteins are one of the most sensitive targets for ROS-mediated posttranslational modifications, and have become the focus of many ROS signaling studies. The electron-rich sulfur atom makes Cys residues the major sites of oxidative modifications within proteins (Akter et al., 2015). It is a well-recognized concept that profiling of ROS/RNS-modified proteins containing Cys can be used to help identify key redox sensors involved in signal transduction pathways (Couturier et al., 2013). Heat stress transcription factors (Hsfs) may also function as ROS-dependent redox-sensors. Hsf proteins contain a DNA binding domain, control the transcription of heat stress associated genes (Baniwal et al., 2004) and are distributed both in the cytosol (mainly in the inactive form) and in the nucleus. Once Hsfs enter the nucleus, they bind to the heat shock elements of the promoters of ROS-sensitive genes, such as the gene encoding APX. There is evidence that certain Hsfs directly sense ROS and control gene expression during oxidative stress (Miller and Mittler, 2006). For example, the transcription of genes encoding the cytosolic peroxidases APX1 and APX2 can be regulated by Hsfs, which in turn can be modulated by ROS (Mazars et al., 2009). Increased expression of Hsfs caused by exogenous H_2_O_2_ was also found to modulate salinity tolerance (Uchida et al., 2002; Wahid et al., 2007). Transgenic *B. napus* plants over-expressing the *Arabidopsis* HEAT SHOCK TRANSCRIPTION FACTORA1b showed enhanced resistance to drought (Bechtold et al., 2013).

#### Signal Transduction, Stress Tolerance and Stress Cross-Tolerance

H_2_O_2_ signaling appears to be integrated with many different signaling networks in plant cells, like Ca^2+^-signaling, protein kinase networks, cellular metabolic networks etc. In some cases, H_2_O_2_ and ROS accumulation was found to precede the activation of signaling, whereas in other cases H_2_O_2_ accumulation was found to be a consequence of signaling. Signal transduction components including protein kinases, such as calcium-dependent protein kinases (CDPKs) and mitogen-activated protein (MAP) kinases have been implicated in stress tolerance as well as cross-tolerance between biotic and abiotic stress responses (Wurzinger et al., 2011). MAPK cascades are important pathways in abiotic stress responses and enable extracellular stimuli to be transduced into intracellular changes (Zhou et al., 2014). A number of cellular stimuli that induce ROS (H_2_O_2_) production can also activate MAPK pathways in multiple cell types (Torres and Forman, 2003; McCubrey et al., 2006). H_2_O_2_- and ROS-responsive MAPKKK, MAPK1, MAPK4, and MAPK6 usually remain highly active under oxidative stress and redox regulation of environmental stress responses. Direct exposure of cells to exogenous H_2_O_2_ leads to activation of MAPK pathways (Dabrowski et al., 2000; Ruffels et al., 2004). The MEKK1 pathway, which was found to be highly active under oxidative stress and induced by unfavorable environmental conditions seems to be the activator of two MAPKKs (MKK1 and MKK2), that in turn activate other MAP kinases (Mittler et al., 2011). MEKK1 is thought to be needed for the activation of MAPK4 by H_2_O_2_ (Mittler et al., 2011). Similarly, MAPK12 was found to be up regulated in response to ABA and H_2_O_2_ application (Mittler et al., 2011). MKP2 is a key regulator of the MPK3 and MPK6 networks that are involved in controlling both abiotic and biotic stress responses (Jammes et al., 2009; Mittler et al., 2011). Zhou et al. (2012) reported the regulatory role of H_2_O_2_ in cold acclimation-induced chilling tolerance in tomato. Cold acclimation induces a modest increase in H_2_O_2_, *RBOH1* gene expression and NADPH oxidase activity that modulates the expression and activity of ROS detoxifying enzymes and ensures stress cross-tolerance. Zhou et al. (2014) further proved the involvement of apoplastic H_2_O_2_ in modulating stress cross-tolerance. Tomato plants pre-treated with mild cold, paraquat (PQ), or drought pre-treatment modulated abiotic stress tolerance by increasing the endogenous level of H_2_O_2_ in the apoplast, which is well correlated with *RBOH1* transcription. An enhanced H_2_O_2_ level was found to modulate the expression of stress and defense-related genes, increase the activities of SOD, APX, CAT, and GR, maintain higher GSH/GSSG ratio and activate MPK1/2. Their findings support the involvement of H_2_O_2_ and MPK1/2 in cross-tolerance in plants. The exogenous application of H_2_O_2_ to *A. thaliana* can activate the MAPK cascades that regulate ROS production and detoxification (Pan et al., 2012). In some cases, MPK3/6 responses to cadmium (Cd) treatment are mediated by the H_2_O_2_-signaling pathway, where MPK3/6 is upregulated after an accumulation of H_2_O_2_ (Liu et al., 2010b). H_2_O_2_ may also be involved in the MAP kinase 8 (MPK8) pathway, since expression of *RBOHD* rapidly decreases via MPK8, resulting in negative regulation of H_2_O_2_ synthesis (Takahashi et al., 2011).

Ca^2+^ one of the most important second messenger in the sophisticated network of plant abiotic stress signaling (Dodd et al., 2010; Wei et al., 2014) and regulation of Ca^2+^ homeostasis is one of the main targets of H_2_O_2_ signaling (Petrov and Van Breusegem, 2012). Regulation of guard cells during stomatal opening is one of the most widely studied processes involving H_2_O_2_-mediated Ca^2+^ signaling and Ca^2+^ is a central signaling component in guard cell responses to stimuli like ABA, ROS and NO (Allen et al., 2000; Young et al., 2006). Ca^2+^ homeostasis also regulates antioxidative defenses in plants. An increase in the concentration of intracellular Ca^2+^ causes efficient detoxification of H_2_O_2_ and involves increased levels of detoxification enzymes, including Ca^2+^-sensitive CAT3. Application of the Ca^2+^ channel blocker LaCl_3_, Ca^2+^ chelator (EGTA) or the calmodulin (CaM) inhibitor (trifluroperazine) to germinating *Amaranthus* seeds, causes a significant reduction in the levels of H_2_O_2_-scavenging enzymes (Bhattacharjee, 2008), strongly supporting the role of Ca^2+^ as a regulator of H_2_O_2_ titer in plant tissues. A rapid increase in cellular Ca^2+^ is considered as one of the earliest responses associated with H_2_O_2_ signaling. Wu et al. (2010) showed that spermidine oxidase-derived H_2_O_2_ regulates pollen membrane hyper-polarization-activated Ca^2+^ channels in order to induce pollen tube growth. In contrast, cytoplasmic Ca^2+^ is able to trigger changes in H_2_O_2_ levels. It is also evident that H_2_O_2_ synthesis requires a continuous cytoplasmic influx of Ca^2+^, which activates NADPH oxidases located at the plasma membrane (Lamb and Dixon, 1997). The Ca–CaM signaling pathway also regulates a number of different target proteins in signaling cascades, including MAP kinases. MAP kinase pathways in turn negatively regulate H_2_O_2_ synthesis by up-regulating the expression of *RbohD*. Thus, CDPKs are involved in tolerance to abiotic stresses (Wei et al., 2014). Treatment of tomato and wheat leaves with H_2_O_2_ increased the expression of CDPKs (Chico et al., 2002; Li et al., 2008). Costa et al. (2010) showed that CAT scavenging of H_2_O_2_ is regulated through a Ca^2+^-dependent pathway in the peroxisomes of *Arabidopsis* guard cells. Recent evidence suggests that RBOH-dependent H_2_O_2_ production might be mediated by Ca^2+^ homeostasis in *Arabidopsis* (Suzuki et al., 2011). In this case, cytoplasmic Ca^+2^ was shown to bind to the EF-hands of the N-terminal region of RBOH and thus promote the activation of RBOH and the production H_2_O_2_ (Takeda et al., 2008). The Ca^2+^ channels and transporters activated by these stimuli form specific Ca^2+^ signatures and changes in these Ca^2+^ signatures are transmitted by protein sensors that preferentially bind Ca^2+^. The binding of Ca^2+^ results in conformational changes in these protein sensors that modulate their activities or their ability to interact with other proteins, and activate the expression of downstream salt-responsive genes through a Ca^2+^ signaling cascade (Rentel and Knight, 2004; Dodd et al., 2010; Kudla et al., 2010; Batistic and Kudla, 2012).

WRKY and zinc finger TFs are both widely involved in the regulation of ROS-related defense genes. It was observed that the ZAT7 and ZAT12 zinc finger proteins of *Arabidopsis* are strongly up regulated by oxidative stress in *apx* knockout mutants in response to H_2_O_2_ and methyl viologen (MV) treatment (Rizhsky, 2004). ZAT10 has a dual role both as an inducer and as a repressor of ROS-responsive genes under salt, drought and osmotic stresses (Sakamoto et al., 2004; Mittler, 2006). ZAT6 positively regulates tolerance to drought, salt, and chilling stress, as well as resistance to bacterial infection, by modulating ROS levels and SA-related gene expression (Shi et al., 2014). Accumulation of NO under stressful conditions was found to initiate defense responses similar to those seen following H_2_O_2_ production (Wang et al., 2014b). NO and H_2_O_2_ are also involved in the stimulation of stomatal closure in *Arabidopsis* in response to ultraviolet-B exposure (He et al., 2005). Removal of H_2_O_2_ with antioxidants or inhibition of its synthesis by inhibiting NADPH oxidase activity prevents NO generation and stomatal closure. Recent evidence supports the idea that H_2_O_2_-induced synthesis of NO might be mediated by MPK6 in *Arabidopsis* (Wang et al., 2010b).

**FIGURE 3:**
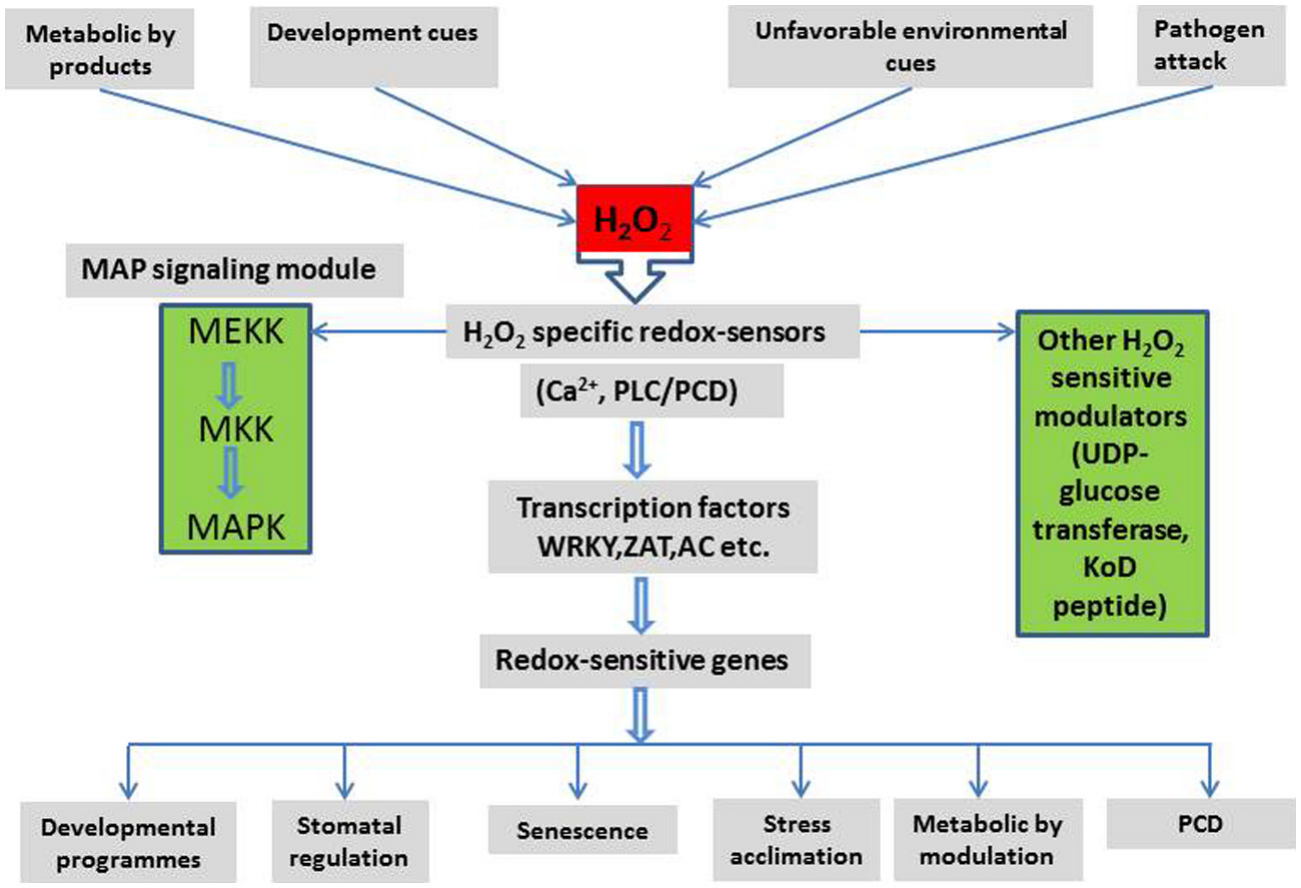
**A schematic representation of major signaling components and their interactions in the H_2_O_2_ signaling network and the possible outcome in plant cells (Detail in text)**.

The links between H_2_O_2_ signaling and other signaling pathways are summarized in **Figure 3**. These demonstrate a complex interaction between H_2_O_2_ signaling, environmental and metabolic cues, their down-stream effects and plant development. It appears that H_2_O_2_ may be involved, in one way or another, in every aspect of plant cell signaling, development, stress responses, and stress tolerance.

#### Gene Expression and H_2_O_2_-Priming and Abiotic Stress Tolerance

Adaptation, the important responses of plants to environmental stresses, is related to environmental stimulation, signal transduction, gene expression, and cellular metabolic adjustment (Pastori and Foyer, 2002). Plants, like other living organisms, sense increased levels of ROS and react with antioxidant mechanisms, finely coordinated, and expressed, to effectively protect the organism from oxidative stress. Modulation of gene expression by H_2_O_2_ has received much attention as H_2_O_2_ is generated in response to a variety of stress stimuli and it is likely to mediate crosstalk between different signaling pathways (Bowler and Fluhr, 2000). H_2_O_2_ modulates the expression of genes involved in ROS control, signal transduction, transcriptional regulation, and protein, carbohydrate, and lipid metabolism, demonstrating the complexity of the transcriptional responses to H_2_O_2_ (Li et al., 2011). A large number of genes involved in stress perception, signal transduction, transcription, defense, and general metabolism have been identified, revealing a highly dynamic and redundant network of genes associated with enzymes involved in ROS-production and ROS-scavenging. In most cases, H_2_O_2_ seems to be positively used by plants to activate multiple stress-responsive genes that help cope with environmental changes. Genes encoding antioxidant enzymes are central players in this network and their function has profound effects in controlling excessive ROS accumulation and cellular redox balance. In contrast, ROS can also negatively regulate the expression of genes encoding antioxidant enzymes, providing a feedback mechanism that can in turn regulate ROS levels, which is a critical component in the modulation of signaling networks (Mylona and Polidoros, 2010). A number of studies have shown that manipulation of plant antioxidant defenses results in cross-tolerance to subsequent exposure of plants to conditions that cause oxidative stress (Neill et al., 2002). Short-term exposure of CAT-deficient mutants to H_2_O_2_ can trigger an increase in tolerance to subsequent severe oxidative stress, indicating that cross-tolerance is mediated by H_2_O_2_ (Vanderauwera et al., 2005). The pivotal role of peroxisomal CAT in decomposing photorespiratory H_2_O_2_ and modulating the signaling role of H_2_O_2_ was recently shown in *Arabidopsis* CAT2-deficient plants (Vandenabeele et al., 2004; Queval et al., 2007). Specifically, photorespiration-generated H_2_O_2_ modulates nuclear transcriptional programs influencing the expression of cytosolic, chloroplastic, and mitochondrial genes, providing additional evidence for the importance of interorganelle communication as part of a plant’s defense response.

Polidoros and Scandalios (1999) showed that high concentrations of H_2_O_2_ rapidly induced *CAT* and *GST1* expression, indicating that oxidative stress directly induces antioxidant responses. They also showed that H_2_O_2_ induced expression of a *GST* gene in the leaves of maize seedlings (Polidoros et al., 2005). GST comprises of a family of nuclear-encoded enzymes involved in cellular detoxification processes following various abiotic stresses, including exposure to xenobiotics and metals (Mylona et al., 2007). Recently, Wang et al. (2013) showed that H_2_O_2_ is involved in the regulation of rice (*O. sativa* L.) tolerance to salt stress where exogenous H_2_O_2_ significantly enhances the activities of APX, CAT, POD, SOD, and G6PDH in a concentration-dependent manner in rice roots. *GPX1* promoter analysis showed that salt induction is mediated via ROS (predominantly H_2_O_2_) in an intracellular process, whereas induction by exogenous H_2_O_2_ involves a different signaling pathway that involves NADPH oxidase. Surprisingly, the promoter of *GPX1* did not respond to exogenous ABA, although *GPX1* transcripts increased in response to ABA in citrus (*Citrus sinensis*) plants. In *Arabidopsis*, it was demonstrated that GPX relays the H_2_O_2_ signal to other signaling molecules, such as ABA (Dietz, 2008). Accumulation of H_2_O_2_ opens Ca^2+^ channels, and induces the generation of Ca^2+^/CaM complexes and the activation of MAPK cascades. TF encoding genes, such as *ZAT10*, *ZAT12,* and *ABI4*, are induced in response to various abiotic and biotic stresses. Transcriptome analysis has shown that some nuclear-encoded TF encoding genes can be induced specifically by the accumulation of a particular ROS (H_2_O_2_), while others are induced by all ROS (Scarpeci et al., 2008). Also a number of TFs have been shown to modulate other antioxidant gene responses. H_2_O_2_ also functions as a major stress signal molecule in plants and the expression of at least 1–2% of *Arabidopsis* genes is now known to be H_2_O_2_-dependent (Desikan et al., 2001). These gene expression studies clearly show that increased cellular H_2_O_2_ levels have a considerable impact on the transcriptome of all plant species, by changing the expression of 100s of genes. These genes are generally involved in cell wall protection, desiccation tolerance, production of ROS scavenging enzymes and DNA damage repair.

#### Ultrastructure Protection by Exogenous H_2_O_2_ Pretreatment and Abiotic Stress Tolerance

Disorganization and/or damage to chloroplast and mitochondrial ultrastructures have been reported in plants in response to excessive accumulation of ROS induced by abiotic stresses (Yamane et al., 2003; Xu et al., 2008; Liu et al., 2010a; Huang et al., 2015). The alteration of chloroplast or mitochondrial ultrastructures was correlated with higher ROS (H_2_O_2_) and MDA accumulation, as well as lower antioxidant enzyme activities under stressful conditions (Xu et al., 2008; Liu et al., 2010a; Huang et al., 2015). H_2_O_2_ pre-treatment induced stress tolerance was associated with higher antioxidant enzyme activities and lower levels of endogenous ROS accumulation (Liu et al., 2010a; Huang et al., 2015). These findings indicate that effective ROS metabolism is essential for maintaining the structural integrity of cellular organelles and keeping them fully functional in plants under abiotic stresses. H_2_O_2_-primed plants can overcome the adverse stress conditions through better ROS metabolism, as well as through the protection of cellular organelles indirectly.

### H_2_O_2_, Hormones and Abiotic Stress Tolerance

**FIGURE 4:**
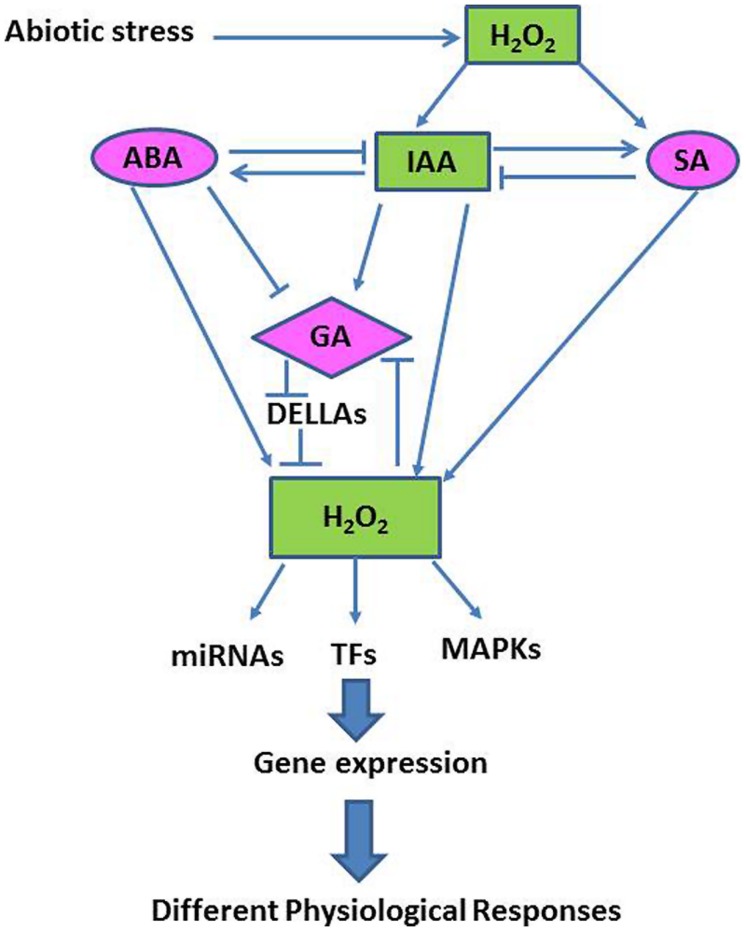
**A simplified schematic model showing interactions between the H_2_O_2_-transduced network and different hormonal signaling pathways in the plant cell (Adapted from Habibi, 2014)**.

Although the precise mechanisms associated with abiotic stress tolerance remain to be fully elucidated, recent studies have found that many abiotic stress responses are coordinated by complex signaling networks, involving both phytohormones and ROS, especially H_2_O_2_ (Bartoli et al., 2013; **Figure 4**). Here we discuss examples demonstrating that major plant hormones interact with H_2_O_2_ and other signaling pathways in modulating plant growth and abiotic stress tolerance.

Abscisic acid is not only an important plant hormone that regulates plant growth and development but also a global regulator that mediates abiotic stress responses. Abiotic stresses can induce an accumulation of ABA (Jia et al., 2002), moreover ABA can induce the production of ROS in plant cells and H_2_O_2_ production in guard cells plays an important role in ABA-mediated stomatal closure (Kwak et al., 2003), while NO production acts downstream of H_2_O_2_ in ABA-induced stomatal closure (Bright et al., 2006). In addition to its function in stomatal closure, ABA also plays crucial role in ROS-mediated stress tolerance. In maize, water stress or ABA treatment influences the accumulation of ROS and increases antioxidant enzyme activities (Jiang and Zhang, 2002), whereas ROS scavengers completely block the ABA-mediated increases in antioxidant activities (Zhang et al., 2006). The expression of NADPH oxidase encoding genes and H_2_O_2_ accumulation in *Arabidopsis* is induced immediately after exposure to salt stress or ABA treatment. H_2_O_2_ accumulation can be suppressed by an inhibitor of NADPH oxidases (DPI) and DPI-treated plants show reduced salt stress tolerance (Zhang et al., 2001; Kwak et al., 2003; Leshem et al., 2007). During ABA-mediated stress responses, MAPK cascades may act both upstream and downstream of ROS production (Zhang et al., 2006; Zhou et al., 2014). MPK9 and MPK12 were identified as downstream factors that integrate ABA-ROS signaling, leading to anion channel activation (Jammes et al., 2009). It was reported that H_2_O_2_ is an essential signal in mediating stomatal closure induced by ABA via activation of Ca-permeable channels in the plasma membrane under a variety of abiotic stresses, such as drought and salinity (Pei et al., 2000; Neill et al., 2002; Kim et al., 2010). In addition, cytokinin-deficient mutants have been found to be tolerant to drought and salinity and have ABA hypersensitivity and reduced levels of ABA (Nishiyama et al., 2011).

Jasmonic acid (JA) and methyl jasmonate (MeJA, a biologically active derivative of JA) are well-established signal molecules involved in a plant’s defense responses and are effective inducers of H_2_O_2_ accumulation in plant cell cultures (Wang and Wu, 2004, 2013). Pre-treatment of *Arabidopsis* and tobacco plants with JA abrogated O_3_-induced H_2_O_2_ accumulation, SA production, and defense gene activation (Overmyer et al., 2000; Rao et al., 2000, 2002). MeJA might also play an important role in signal transduction in grape cells, regulating the levels of NO and H_2_O_2_ and enhancing the activities of enzymes involved in phytoalexin synthesis (Wang et al., 2012). Like ABA, MeJA can induce stomatal closure mediated by ROS-dependent signaling pathways (Suhita et al., 2004). JA can also interact with ABA, ethylene (ET) and SA in oxidative stress triggered by water deficiency (Brossa et al., 2011). Increased JA accumulation under drought was found to increase the transcript abundance and activities of APX, GR, MDHAR, DHAR, and L-galactono-1,4-lactone dehydrogenase (GalLDH), as well as enhancing the contents of AsA and GSH (Shan and Liang, 2010). During water stress, transient accumulation of JA is needed for ABA increases in citrus roots (de Ollas et al., 2013).

Salicylic acid (SA) plays an important role in plant responses to various types of environmental stresses, including drought, low temperature and high salinity (Kang et al., 2014; Miura and Tada, 2014). Under stress, the levels of endogenous SA and H_2_O_2_ increase in plant cells. However, the levels H_2_O_2_ degrading enzymes, such as CAT and APX, can be suppressed by SA treatment (Yuan and Lin, 2008). Previous studies have shown that H_2_O_2_ alone is not sufficient to trigger antioxidant defense responses and cell death in cell cultures (Chen et al., 1993). However, a SA-deficient transgenic *Arabidopsis* line overexpressing salicylate hydroxylase (*NahG*) gene exposed to O_3_ accumulated more H_2_O_2_ compared with non-transgenic plants, yet failed to efficiently induce defense genes, such as *GST1* and *GPX*, and perturb the redox state of glutathione (Rao et al., 2002). This indicates that H_2_O_2_ alone is not sufficient to trigger defense responses, and SA is required to maintain the cellular redox state and potentiate defense responses in O_3_-exposed plants (Rao and Davis, 1999). Pre-treatment with SA causes ROS accumulation (Mittler, 2002; Harfouche et al., 2008). ROS production mediated by salicylhydroxamic acid (SHAM)-sensitive guaiacol peroxidases was induced by SA in guard cells, which may be independent of the ABA pathway (Mori et al., 2001; Khokon et al., 2011). Other enzymes inhibited by SA, such as APX, CAT, and carbonic anhydrase, are also involved in scavenging ROS (Chen et al., 1993; Conrath et al., 1995; Durner and Klessig, 1995; Slaymaker et al., 2002). SA and ROS, mainly H_2_O_2_, have been proposed to form a self-amplifying feedback loop in response to abiotic and biotic stresses (Vlot et al., 2009).

Auxins have been shown to play an important role in stress-related hormonal networks. They can indirectly modulate ROS homeostasis by affecting the stability of DELLA proteins (Fu and Harberd, 2003; Weiss and Ori, 2007; Paponov et al., 2008) or directly by inducing ROS detoxification enzymes, such as GSTs and quinine reductases (Laskowski et al., 2002). Analysis of the TRANSPORT INHIBITOR RESPONSE 1 (TIR1)/AUXIN SIGNALLING F-BOX PROTEINS (AFB) auxin receptors shows that these genes are involved in tolerance to oxidative stress by regulating H_2_O_2_, antioxidant enzymes, antioxidant levels and the chlorophyll content of plants (Iglesias et al., 2010). In addition, auxin-induced H_2_O_2_ production acts as a signal for the stomatal opening response (Song et al., 2006). H_2_O_2_ is also involved in auxin-induced root geotropism (Joo et al., 2001). Any exogenous or endogenous stimulus that perturbs cellular redox balance can activate auxin homeostasis because NADPH oxidase-dependent ROS production influences polar auxin transport (Joo et al., 2001, 2005).

Gibberellic acids (GAs) mediate growth in response to environmental signals by relieving the constraints on gene expression imposed by a family of growth-repressing regulators, the DELLA proteins (Peng et al., 1999; Harberd et al., 2009). Moreover, the *Arabidopsis* GA-deficient *ga1-3* mutants, that have higher levels of DELLA proteins, are much less susceptible to ROS-dependent cell death (Achard et al., 2008). Stress (drought or high salt) conditions enhance ABA/GA ratios favoring DELLA protein accumulation and lower ROS levels (Finkelstein et al., 2008; Considine and Foyer, 2014). ABA can decrease ROS production in rice seeds, leading to a repression of ascorbate and GA accumulation (Ye et al., 2012). Recently, it was reported that ROS are involved in GA/ABA signaling in barley aleurone cells (Ishibashi et al., 2012). In addition, GA-stimulated *Arabidopsis* gene *GASA14* regulates leaf expansion and abiotic stress tolerance by modulating ROS accumulation (Sun et al., 2013). The overexpression of *GASA4* suppressed ROS accumulation in transgenic plants and enhanced their tolerance to NO (Rubinovich and Weiss, 2010).

Ethylene can induce ROS generation and H_2_O_2_ can stimulate the expression of ET biosynthesis and responsive genes (Vandenabeele et al., 2003). The PCD pathways occurring in leaf abscission depends on NADPH oxidase-dependent H_2_O_2_ generation triggered by ET (Sakamoto et al., 2008). Moreover, stomatal closure also requires the integrated cooperation of H_2_O_2_ and ET. In *Arabidopsis*, the ETHYLENE RESPONSE FACTOR1 (ERF1) is greatly induced by high salinity and drought stress (Cheng et al., 2013). Induction of the salt stress response in *Arabidopsis* required both JA and ET signaling, but was inhibited by ABA. Transgenic plants overexpressing ERF1 showed higher salt and drought tolerance and less water loss due to transpiration (Cheng et al., 2013).

From the above discussion, it is evident that ROS production and associated redox processing are an integral part of hormone regulation and functioning in the control of plant abiotic stress responses and tolerance. Further understanding of H_2_O_2_-hormone-antioxidant interactions will help to facilitate the development of plants tolerant to various abiotic stresses.

## Conclusion and Future Perspectives

H_2_O_2_ priming represents a fruitful area of future research, which should help plant scientists explore the molecular mechanisms associated with abiotic stress tolerance and promote a more environmental friendly sustainable agriculture. Plants have mechanisms, particularly under environmental stress, to utilize ROS, especially H_2_O_2,_ for signaling purposes that confer acclamatory stress tolerance through the modulation of osmotic adjustment, ROS detoxification, photosynthetic C fixation and hormonal regulation. A large number of studies have suggested that pre-treatment of seeds or seedlings with H_2_O_2,_ or the combined application of H_2_O_2_ and abiotic stress, induces an inductive pulse that helps to protect plants under abiotic stresses by restoration of redox-homeostasis and mitigation of oxidative damage to membranes, proteins and lipids and by modulating stress signaling pathways (**Figures 2–4**), however, the mechanisms for this are not well established. Many researchers have suggested a central role for H_2_O_2_ in intracellular and systemic signaling routes that increase tolerance and acclimation to abiotic stresses bur how H_2_O_2_ is sensed by plant cells is still a mystery, and numerous factors are still being considered with respect to their roles in H_2_O_2_-induced signal transduction and development of responses to oxidative stress. Recent findings have shown that effective ROS signaling may require an increased flux of antioxidants, notably those that are thiol-dependent. With respect to signal transduction, ROS can interact with other signaling pathways, such as the activation of NADPH oxidase dependent or monomeric G protein; lipid-derived signals; induction of MAPK; redox sensitive TFs; regulation of Ca^2+^; and plant hormone signal transduction. An understanding of the H_2_O_2_ physiology of plants, particularly H_2_O_2_ sensing and the identification of the components of H_2_O_2_ signaling network and H_2_O_2_ cross-talk with other growth factors, is necessary and is of great importance if we are to improve the performance of crop plants growing under conditions that cause abiotic stress.

Identification of the genetic network and of the downstream reactions modulated by H_2_O_2_ originating from specific organelles remains a future challenge. Additionally, one of challenges in H_2_O_2_ research is to identify specific H_2_O_2_ receptors and to establish how the cell is able to decode endogenous H_2_O_2_ signals and discriminate between different stimuli giving rise to a very specific defense response. Utilization of genetically engineered plants that suppress H_2_O_2_ generation or increase H_2_O_2_ production and the isolation of H_2_O_2_-signaling mutants will be invaluable in elucidating further the biological roles of H_2_O_2_ in specific cells and in the response to various abiotic stressors that cause oxidative damage. Further proteomic, metabolomic and transcriptomic studies will provide further insights into the responses of cells to H_2_O_2_. Future research is likely to find new signaling roles for H_2_O_2_ in regulating ROS scavenging systems with respect to modulating abiotic stress tolerance. A better understanding of H_2_O_2_ and its role in regulating ROS scavenging systems will be valuable in helping produce crop plants with greater levels of tolerance to oxidative stress using traditional plant breeding or biotechnological approaches. Understanding the subtle and sensitive mechanisms plants use to fine-tune H_2_O_2_ titer and the associated signaling cascades may hold the key to improving agriculture in the future.

## Conflict of Interest Statement

The authors declare that the research was conducted in the absence of any commercial or financial relationships that could be construed as a potential conflict of interest.
